# Serotonin Syndrome From Duloxetine Monotherapy: A Case Report

**DOI:** 10.7759/cureus.40933

**Published:** 2023-06-25

**Authors:** Liumin Li, Qu Mi

**Affiliations:** 1 Department of Psychiatry, Huzhou Third People's Hospital, the Affiliated Hospital of Huzhou University, Huzhou, CHN

**Keywords:** depressive disorder, adverse drug reactions, case report, serotonergic drug, duloxetine, serotonin syndrome

## Abstract

Serotonin syndrome is a rare adverse reaction induced by serotonergic drugs. Most instances of the syndrome occur in the context of a serotonergic drug overdose and the combined use of monoamine oxidase inhibitors and other related drugs. We report a case of serotonin syndrome caused by duloxetine alone in an elderly Asian woman and review the literature. A 69-year-old woman was admitted to the hospital due to two months of irritability and reduced energy. She was diagnosed with depressive disorder in the outpatient department and took sertraline 75mg for one month, but there was no significant improvement of symptoms. After admission, sertraline was switched to duloxetine. After taking duloxetine 60mg, the patient developed confusion, inducible clonus, diaphoresis, tremor, hyperreflexia, and increased body temperature and blood pressure. Reviewing her history of drug exposure, physical examination, and associated laboratory tests, we ruled out other possible diseases and established a serotonin syndrome diagnosis. The symptoms and signs associated with serotonin syndrome disappeared within two days after treatments with hydration and diazepam and the withdrawal of duloxetine. Physicians should be watchful for serotonin syndrome, a rare, but in severe cases life-threatening, adverse drug reaction. It may occur with duloxetine monotherapy even at therapeutic doses.

## Introduction

Serotonin syndrome is a rare adverse reaction induced by serotonergic drugs, whose severity can range from mild to life-threatening [1]. Typical symptoms of serotonin syndrome include neuromuscular abnormalities, altered states of consciousness, and hyperactivity of the autonomic nervous system, resulting from excessive activation of serotoninergic function in the central and peripheral nervous system [2-4]. Many types of drugs and drug interactions are associated with serotonin syndrome, most of which occur in the context of a serotonergic drug overdose and the combined use of monoamine oxidase inhibitors and other related drugs [5]. Cases of serotonin syndrome caused by monotherapy at therapeutic doses are rare but occasionally reported [6-9]. Duloxetine, a selective serotonin and noradrenaline reuptake inhibitor, was not included in the list of drugs that may cause serotonin syndrome [5]. However, three cases of serotonin syndrome have been reported in patients using duloxetine alone at therapeutic doses [8-10]. We report a case of serotonin syndrome caused by duloxetine monotherapy in an elderly Asian woman.

## Case presentation

Chief complaints

A 69-year-old woman was admitted to the hospital due to two months of irritability and reduced energy.

History of present illness

The patient diagnosed with depressive disorder in the outpatient department took sertraline 75mg for one month. She was compliant with medication, with no instances of overdose or missed doses. She was admitted to the hospital because of the poor outcome of outpatient treatment. The total score of the 17-item Hamilton Depression Rating Scale (HAMD) at admission was 18. The patient had already taken 75mg of sertraline on the morning of admission. On the second day of hospitalization, sertraline was discontinued and the patient started taking 30mg of duloxetine. On the third day of hospitalization, the patient took 60mg of duloxetine. Subsequently, the patient developed symptoms of confusion, slow responsivity, and unstable walking.

History of past illness

She had a history of hypertension only. She took levamlodipine 2.5mg daily, and her blood pressure was controlled steadily. The patient denied using any other drugs except levamlodipine and antidepressants.

Personal and family history

She had no history of alcohol and addictive substance abuse and had no relevant family history.

Physical examination

After taking duloxetine 60mg, her blood pressure was 152/85 mmHg, heart rate was 121 beats/min, respiratory rate was 22 breaths/min, and temperature was 38.4 ℃. On physical examination, we discovered hyperreflexia (more evident in the lower extremities than upper extremities), myoclonus, tremors, mydriasis, and diaphoresis. No additional extrapyramidal signs were found.

Laboratory examinations

On the first day of admission, the patient showed obvious hyponatremia, hypochloremia, and hypokalemia (Table 1). Results from a routine blood test and assessments of inflammatory indicators, liver and kidney function, and thyroid function were all within the normal range. After receiving electrolyte supplementation, her electrolyte levels became normal by the third day after admission. At this time, leukocytes and neutrophils were elevated (Table 1). However, she lacked symptoms of infection, and imaging examinations did not show signs of infection.

**Table 1:** Selected indicators after admission ^1^Day 1: The patient stopped using sertraline after admission. ^2^Day 3: The patient took duloxetine 60 mg in the morning and subsequently discontinued duloxetine and received appropriate therapy. ^3^Day 6: The symptoms and signs associated with serotonin syndrome, such as confusion, inducible clonus, and tremors, disappeared after treatment. ^4^Month 2: The patient was discharged with a HAMD score of 5. ALT: Alanine aminotransferase; AST: aspartate aminotransferase; NT-proBNP: N-terminal pro-brain natriuretic peptide; cTnT: cardiac troponin T; TSH: thyroid stimulating hormone; FT3: free tri-iodothyronine; FT4: free thyroxine; HAMD: 17-item Hamilton Depression Rating Scale

	Day 1^1^	Day 3^2^	Day 6^3^	Month 1	Month 2^4^	Reference
Routine blood test						
Leukocytes	7.82	10.43	4.28	3.77	4.55	3.5-9.5 × 10^9^/L
Neutrophils	6.28	7.98	3.52	2.37	2.98	1.80-6.30 × 10^9^/L
Lymphocytes	0.73	1.66	0.52	0.88	1.13	1.10-3.20 × 10^9^/L
Hemoglobin	134	137	118	114	116	115-150 g/L
Platelets	271	283	225	271	215	125-350 × 10^9^/L
Inflammatory indicators						
C-reactive protein	0.73	1.24	1.10	0.78	1.50	< 6.00 mg/L
Electrolytes						
K^+^	2.71	4.02	4.06	3.98	4.14	3.5-5.3 mmol/L
Na^+^	131.1	128.9	132.2	141.5	143.0	137-147 mmol/L
Cl^-^	85.6	92.5	92.0	102.0	106.6	99-110 mmol/L
P	0.77	1.04		1.15	1.07	0.85-1.51 mmol/L
Ca^2+^	2.18	2.30		2.14	2.24	2.11-2.52 mmol/L
Liver function						
ALT	19.8	28.3	30.0	27.5	20.8	7-40 U/L
AST	25.6	30.7	39.0	20.2	23.2	13-35 U/L
Albumin	42.2	47.8	36.9	38.5	41.1	40.0-55.0 g/L
Renal function						
Serum creatinine	52.2	50.3	56.9	58.6	67.4	45.0-84.0 μmol/L
Urea	3.76	5.43	6.00	4.87	6.14	3.10-8.80 mmol/L
Uric acid	155.6	119.0		256.2	166.4	142.0-339.0 μmol/L
Coagulation function						
Prothrombin time	10.4	10.0		9.8	10.2	9.8-12.1 s
Activated partial thromboplastin time	25.2	26.4		23.8	25.8	25.0-31.3 s
Thrombin time	18.3	18.6		18.0	18.8	14.0-21.0 s
D-dimer	0.27	1.24		0.51	0.34	< 0.55 mg/L
Heart function						
NT-pro BNP		375.6				
cTnT	11.76	12.83	9.36	8.14	12.62	<14 pg/mL
Myoglobin	452.30	96.70	30.95	22.27	24.44	25.0-58.0 ng/mL
Blood gas analysis						
PH		7.441				7.350-7.450
PaCO2		36.7				35.0-45.0 mmHg
PaO2		99.2				80.0-110.0 mmHg
Actual bicarbonate radical		23.2				21.4-27.3 mmol/L
Standard bicarbonate radical		24.6				21.3-24.8 mmol/L
Thyroid function tests						
TSH	1.240					0.270-4.200 μIU/mL
FT3 free tri-iodothyronine	2.52					2.00-4.40 pg/mL
FT4 free thyroxine	1.95					0.93-1.70 ng/dL
Other indicators						
Ferritin	203.4					13.0-150.0 ng/mL
Folate	5.51					2.70-34.00 ng/mL
HAMD	18				5	
Screenings for infectious diseases such as hepatitis A, B, C, E, syphilis, and AIDS were all negative.						

Imaging examinations

Her electrocardiogram (ECG) showed sinus tachycardia, and computed tomography (CT) of her head and chest, magnetic resonance imaging (MRI) of her head, and electroencephalography did not reveal any obvious abnormalities.

Final diagnosis

Considering the patient's medication history, altered state of consciousness, abnormal autonomic nervous system function, and neuromuscular abnormalities, we diagnosed the patient with serotonin syndrome.

Treatment

Duloxetine was discontinued, intravenous fluid hydration was applied to accelerate drug metabolism, and a nutritional solution was administered through a nasal feeding tube. Moreover, an intravenous drip of diazepam 5mg was administered for tremors and confusion.

Outcome and follow-up

In the next 24 hours, the patient's condition improved rapidly. The patient's state of consciousness returned to normal, and she could normally communicate with the doctor, with alleviation of myoclonus and stiffness. Without the use of additional antihypertensive and antipyretic drugs, her temperature, blood pressure, and heart rate returned to normal. A slight limb tremor remained but disappeared within two days. When talking about the previous day's experiences, the patient reported that she did not remember most of them but remembered seeing many frightening images. In the subsequent reexamination, the levels of leukocytes and neutrophils returned to normal.

Considering that the patient's depressive disorder first appeared after a family event and was moderate in severity, she subsequently received cognitive behavioral therapy. Two months later, she was discharged with a HAMD score of 5. She has exhibited a stable mood for four months since the follow-up.

## Discussion

Among several different types of diagnostic criteria for serotonin syndrome, decision rules based on Hunter criteria are commonly used for their simplicity and higher sensitivity (84%) and specificity (97%) [4,11,12]. The current patient took 30mg of duloxetine before the onset of symptoms, which was subsequently increased to 60mg. The 60mg dose was followed by confusion, inducible clonus, diaphoresis, tremor, hyperreflexia, and an increase in body temperature and blood pressure. Based on a review of her medication history (no antipsychotic medications, no substance abuse or withdrawal), physical examination, and associated laboratory tests (no abnormalities were found in the CT of the head and chest; blood inflammation indices were normal), we ruled out other possible diseases such as neuroleptic malignant syndrome, encephalitis, and cerebral vasculitis, and established the diagnosis of serotonin syndrome [1,13]. The diagnosis was further confirmed by the rapid improvement of the patient's condition after treatment with hydration and diazepam and withdrawal of duloxetine [5,14]. Early recognition and appropriate management prevented the syndrome from progressing to a severe form and prevented severe complications such as shock, rhabdomyolysis, seizures, and disseminated intravascular coagulopathy [5].

We determined that duloxetine alone is the drug that caused this case of serotonin syndrome. Levamlodipine had been used for antihypertensive therapy, and sertraline was replaced with duloxetine for antidepressant treatment after admission. There was no obvious pharmacological interaction between levamlodipine and sertraline or duloxetine. Serotonin syndrome-related symptoms appeared after the administration of duloxetine, more than 24 hours after the last dose of sertraline, and did not appear during sertraline treatment. The elimination half-life of sertraline is 24 hours. Desmethylsertraline, the major metabolite of sertraline, has a negligible clinical effect, with a serotonin reuptake inhibition capacity of only 5-10% of that of sertraline [15]. Both sertraline and desmethylsertraline are inhibitors of CYP2D6 enzymes and have no significant inhibition on CYP3A3/4, CYP1A2, and CYP2C19 at the therapeutic dose [16,17]. However, studies have shown that CYP2D6 enzyme inhibitors have a smaller effect on duloxetine metabolism than CYP1A2 inhibition, and there is no need for dose adjustment when using duloxetine [18]. Considering that the patient had not used other related drugs such as opiate analgesics, weight-reduction agents, drugs of abuse, or herbal products, duloxetine monotherapy is the most likely triggering drug [5].

To date, three serotonin syndrome cases caused by duloxetine monotherapy have been reported. Hansbauer et al. reported a 41-year-old white male with coeliac disease who developed serotonin syndrome after administration of duloxetine 60mg [8]. The authors considered that coeliac disease elevated plasma 5-HT levels and, together with duloxetine, promoted the syndrome's development. Gelener et al. reported a case of serotonin syndrome in a 29-year-old woman after taking duloxetine 30mg [10]. The third case was a 24-year-old female who presented with serotonin syndrome after 10 days of continuous administration of duloxetine 30mg [8].^ ^The syndrome did not resolve completely in this patient until 14 days after discontinuation of duloxetine and the combined usage of cyproheptadine and benzodiazepines. Among these three cases, the 41-year-old white male patient experienced severe systemic inflammatory response syndrome and recovered after receiving ICU treatment for a period of time. The liver and kidney functions of the other two patients were normal. Serotonin syndrome with duloxetine monotherapy can occur in all ages, and we add this case of a 69-year-old Asian woman caused by duloxetine 60 mg, which completely resolved within two days of treatment.

It has been suggested that a serotonin transporter polymorphism may partially explain the differences in the risk of developing serotonin syndrome between individuals at the genetic level [19]. The lack of related genetic tests and plasma duloxetine concentration in this patient is a limitation of this case. In addition, the patient took duloxetine after a 24-hour discontinuation of sertraline, during which sertraline had not been fully metabolized. Therefore, we cannot rule out the possibility that the combination of sertraline and duloxetine contributed to serotonin syndrome.

## Conclusions

Cases of serotonin syndrome caused by single duloxetine at therapeutic doses are rare. When a patient has a history of serotonergic drug exposure and presents with a clinical triad of neuromuscular abnormalities, altered states of consciousness, and hyperactivity of the autonomic nervous system, serotonin syndrome should be considered even if only a single serotonergic drug was used. A serotonin transporter polymorphism may account for differences between individuals in the risk of acquiring the syndrome. Serotonin syndrome occurs after exposure to or increased doses of a serotonergic drug. With timely discontinuation and appropriate treatment, most patients with mild to moderate disease can be relieved quickly.
